# Chemoradiotherapy followed by surgery versus surgery alone in esophageal cancer patients: is it time for additional evidence?

**DOI:** 10.1186/1477-7819-9-41

**Published:** 2011-04-19

**Authors:** Stefano Cafarotti, Alfredo Cesario, Venanzio Porziella, Stefano Margaritora, Pierluigi Granone

**Affiliations:** 1 Division of General Thoracic Surgery, Catholic University, Rome, Italy; 2 Unit of Clinical and Molecular Epidemiology, IRCCS San Raffaele Pisana, Rome, Italy

## Abstract

Recent efforts to improve survival in patients with locally advanced esophageal carcinoma have combined both systemic and local therapy. However, the role of neoadjuvant chemoradiotherapy in technically operable IIa-III esophageal carcinoma is still unresolved.

## Findings

We have read with interest the report from Hurmuzlu and coll [1] on the outcome of induction therapy (IT) plus surgery versus surgery alone in locally advanced operable esophageal cancer (OC).

The report is of great speculative interest given the consistently poor prognosis of OC whatever the therapeutic strategy adopted: so far, in fact, there is no general consensus on the appropriate treatment for such a dreadful condition. Specifically, the role of chemo-radiotherapy administered pre-operatively in resectable cstage IIa-III OC is still discussed.

Scarce data are available from the literature and these are not consistent. In fact some experiences [2,3] conclude with positive recommendations to adopt the tri-modality approach and others [4] conclude with opposite position: that IT should not be adopted in OC that are resectable following the clinical staging assessment. As already advocated by Pereira [5], the indication for IT for resectable OC remains largely not evidence-based substantially due to methodological biases in the trials that can be summarised as follows: different tumour stages included, no standardized preoperative diagnostic procedure and, last but not least, the great heterogenity of surgical treatment.

In this scenario of substantial absence of a large base of methodologically correct evidence and agreed guidelines we consider the results from [1] of significant clinical value and concur in advocating for further evidence stemming from large scale prospective randomised trials. Ideally, these should be designed valuing the past experiences to address the methodological biases with the precise task to assess whether IT should be administered before surgery in resectable OC. These trials should: a) distinguish between hystologies (squamous cell vs adenocarcinoma); b) include an optimal pre-operative staging with EUS, high quality CT and PET scan to assess the extent of the loco-regional disease and exclude distant metastases and c) include a standardized surgical treatment with extended lymph node dissection. Only by such trials the role of IT in the treatment of OC can be cleared. More convincing arguments, in fact, need to support any proposed change in clinical behaviour.

## Competing interests

The authors declare no conflicts of interest

## Authors' contributions

All authors read and approved the final manuscript.
